# Blood vessels as targets in tumor therapy

**DOI:** 10.3109/03009734.2012.660550

**Published:** 2012-04-19

**Authors:** Lena Claesson-Welsh

**Affiliations:** Uppsala University, Department of Immunology, Genetics and Pathology, Rudbeck Laboratory, Uppsala, Sweden

**Keywords:** Angiogenesis, cancer, FGF, PDGF, VEGF

## Abstract

The landmark papers published by Judah Folkman in the early 1970s on tumor angiogenesis and therapeutic implications promoted the rapid development of a very dynamic field where basic scientists, oncologists, and pharmaceutical industry joined forces to determine the molecular mechanisms in blood vessel formation and find means to exploit this knowledge in suppressing tumor vascularization and growth. A wealth of information has been collected on angiogenic growth factors, and in 2004 the first specific blood vessel-targeted cancer therapy was introduced: a neutralizing antibody against vascular endothelial growth factor (VEGF). Now (2011) we know that suppression of tumor angiogenesis may be a double-edged sword and that the therapy needs to be further refined and individualized. This review describes the hallmarks of tumor vessels, how different angiogenic growth factors exert their function, and the perspectives for future development of anti-angiogenic therapy.

## Blood vessels in health and disease

Blood is carried by a hierarchical network of vessels lined by a single layer of endothelial cells (EC). Circulation of blood allows delivery of oxygen to the tissues (1), which is essential for the generation of energy by the mitochondria. Nutrients are taken up by the blood passing through thin-walled capillaries in the intestinal tract, and storage of nutrients and detoxification of the blood are mediated by hepatic and renal circulation, respectively. The ECs deposit a basement membrane composed of fibronectin, collagen IV, laminins, and heparan sulfate proteoglycans (HSPG) (2), which directly or indirectly influences diverse processes such as cell differentiation, attachment, migration, polarization, guidance, and survival. Vessels are surrounded by smooth muscle cells in a manner dependent on their size and position vis-à-vis the heart; arteries display a muscular coat that regulates the vascular tone, whereas capillaries are more sparsely supported by specialized mesenchymal cells denoted pericytes.

Formation of new vessels, angiogenesis, is critical in growing tissues. The principles of blood vessel formation differ dependent on whether blood vessels form during embryogenesis (denoted vasculogenesis), during physiological processes such as wound healing or growth of the endometrium (denoted regulated angiogenesis), or during pathologies such as inflammation and cancer (denoted pathological, or dysregulated angiogenesis). In addition, vessels may form through splitting, in a process called intussusception (3), or may be derived from circulating, bone-marrow-derived progenitors (denoted adult or postnatal vasculogenesis) (4).

**Figure 1.**
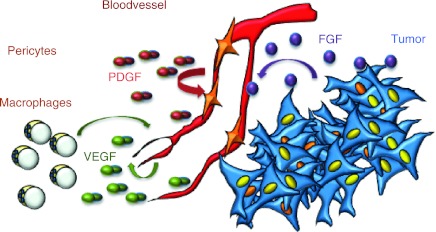
Production of VEGF, FGF, and PDGF in the tumor microenvironment. VEGF is produced by most cells in the tumor microenvironment, including endothelial cells and macrophages. It exerts its effects on endothelial cells; occasionally, tumor cells may express VEGF receptors and respond to VEGF. PDGF is produced by endothelial cells and serves to attract pericytes to embed the newly formed vessel; however, in the tumor, pericytes as a rule fail to wrap tightly around the endothelial cells. FGF is produced by tumors cells and may act directly on tumor vessels but not on endothelial cells in healthy tissues.

Several features distinguish vessels formed during physiological processes from those in pathologies such as cancer. Healthy vessels are arranged in a hierarchical manner (arteries, capillaries, and veins), whereas the tumor vasculature is disorganized and morphologically abnormal. Healthy vessels are moreover perfused, whereas tumor vessels display partial or complete occlusions leading to poor blood flow (5). Pericytes, which depend on production of platelet-derived growth factor (PDGF) by endothelial cells, embed healthy capillaries. In contrast, tumor vessels often have a scarce coat of loosely associated pericytes (6). Moreover, the vascular basement membrane may be discontinuous. Tumor vessels are leaky, in part due to the deficient perivascular support, but also due to abundant expression of vascular endothelial growth factor (VEGF), also denoted vascular permeability factor (VPF), or inflammatory cytokines in the growing tumor (5). The leakiness leads to a build-up of interstitial pressure and impaired drug delivery. The edema may in itself aggravate the condition e.g. for patients suffering from brain tumors such as glioblastoma multiforme. Production of other growth factors such as fibroblast growth factor (FGF) by tumor cells may further promote excess growth and dysregulation of the vasculature. Thus, a number of growth factors exert their effect in the tumor microenvironment in a manner that promotes progression of the disease (Figure 1).

## Growth factors in regulation of blood vessel formation

Growth factors are major stimulators of angiogenesis. A large number of second messengers may modulate, positively or negatively, their downstream effect. Studies on gene-targeted animals have been instrumental in deducing the role of different growth factors, especially during embryonic development. Below, I will discuss the features of a number of key angiogenic growth factors.

### Vascular endothelial growth factors (VEGFs)/vascular permeability factor (VPF)

#### Ligands

VEGF denotes a family of five structurally related mammalian ligands: VEGFA, VEGFB, VEGFC, VEGFD, and placental growth factor (PlGF). VEGF was originally identified as vascular permeability factor (VPF) (7). The VEGFs are composed of covalently linked homodimers (8), but there are also naturally occurring heterodimers, e.g. of VEGFA and PlGF (9). Alternative splicing and processing generate further diversity. Related polypeptides from other species include the VEGFEs, which are open reading frame(s) in parapoxvirus, and the VEGFFs, found in snake venom. VEGFA is up-regulated in hypoxia, as shown originally by Eli Keshet and co-workers (10).

#### Receptors

**Figure 2.**
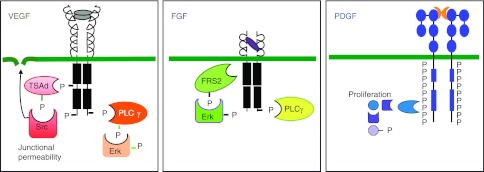
Properties of VEGF, FGF, and PDGF receptors and their signaling. Schematic outline of receptors is shown. All three VEGF receptors are organized into an extracellular domain with seven Ig-like folds and a short kinase insert. Tyrosine phosphorylation sites have been identified at some positions. Key features of VEGFR2 signaling are the binding of the adaptor molecule TSAd to the kinase insert phosphorylation site Y951 and regulation of Src activation followed by opening of adherens junctions. A C-terminal phosphorylation site at Y1175 allows binding of PLCγ and signaling in the Erk pathway. FGF receptors are composed of three Ig-like loops extracellularly, a very short kinase insert, and signaling via FRS2, which associates to the FGFR without involvement of tyrosine phosphorylation sites. PLCγ is an important downstream signal transducer in FGF biology. PDGF receptors have five extracellular Ig-like loops and a long kinase insert. The PDGF receptors become phosphorylated at very many tyrosine residues in response to ligand binding and induce formation of several long signaling chains. Erk = extracellular regulated kinase; FRS2 = FGF receptor substrate 2; P = phosphate; PLCγ = phospholipase Cγ; TSAd = T cell specific adaptor.

VEGF ligands bind to three related receptor tyrosine kinases, denoted VEGFR1, VEGFR2, and VEGFR3. VEGFA, VEGFB, and PlGF bind to VEGFR1. VEGFA, VEGFC, and VEGFD bind to VEGFR2; and VEGFC and VEGFD bind to VEGFR3. In cells coexpressing VEGFR2 and VEGFR3, VEGFC and VEGFD will induce heterodimers of the receptors. VEGFRs show a similar organization with an extracellular, ligand-binding domain composed of seven immunoglobulin (Ig)-like loops, a transmembrane domain, a juxtamembrane domain, a split tyrosine kinase domain, and a C-terminal tail (Figure 2). There is an overall pattern of VEGFR1 expression in monocytes and macrophages, VEGFR2 in vascular endothelial cells, and VEGFR3 in lymphatic endothelial cells. However, clearly the expression of the receptors is dynamic, and, for example, VEGFR3 is expressed in vascular endothelial cells engaged in active angiogenesis (11). Whereas ECs in the stable vasculature weakly express VEGFR2, the receptor is up-regulated and expressed at high levels during physiological or pathological angiogenesis (e.g. in tumors) (12).

VEGFs also bind to a range of accessory molecules, which occasionally are denoted VEGF receptors. These include heparan sulfate proteoglycans (HSPGs)/heparin, the neuropilins, and integrins (13). As these molecules are devoid of intrinsic catalytic activity, it is more appropriate to use the designation ‘VEGF co-receptor’. The VEGF co-receptors are essential for VEGF biology and direct the cellular response in a fundamental way. Thus, HSPGs and the mast cell-secreted heparin bind growth factors in a charge-dependent manner and mediate efficient presentation to the cognate receptor. HSPGs may also bind directly to the receptor such as VEGFR2 (14). By creating local growth factor gradients, HSPGs provide guidance cues for new vessels. Neuropilins (neuropilin1 and neuropilin2), bind semaphorins, axon-guidance molecules, and VEGF family members to non-overlapping extracellular domains (15). NRP1 regulates VEGF-induced vessel sprouting and branching. Moreover, NRP1 binds to the adaptor GIPC/synectin and thereby directs VEGFR2 internalization. Integrins engage in complex formation with VEGF receptors and direct downstream signaling and endothelial migration.

#### Lessons from genetic models

Gene targeting of VEGFA and VEGFR2 results in similar phenotypes with arrest in EC differentiation and embryonic death around embryonic day E8.5. Clearly, VEGFA/VEGFR2 function is a strict prerequisite for EC development, EC survival, and for regulation of vascular permeability in the adult (13).

In contrast to VEGFA, elimination of either VEGFB or PlGF is compatible with embryonic development. Interestingly, the ligands for VEGFR1 have very different functions. VEGFB has been assigned a specific role in revascularization of the ischemic myocardium (16) and, importantly, in fatty acid transport over the endothelium to the target tissue (17). In contrast, PlGF has been more generally implicated in regulation of pathological angiogenesis. Thus, PlGF-deficient animals develop normally, but, due to a reduced angiogenic response, tumor growth and inflammatory processes are attenuated (18). Gene targeting of VEGFR1, which binds VEGFA, VEGFB, and PlGF, leads to embryonic death around E9.5, due to excess proliferation of EC and poor organization of cells to form lumenized vessels (19). It is noteworthy that VEGFR1 is produced not only as a full-length receptor but also as a soluble extracellular domain. This soluble form binds VEGFA with high affinity and has been suggested to serve as a trap, immobilizing VEGFA and preventing it from binding to VEGFR2 (20). As mice expressing a truncated VEGFR1, lacking the intracellular tyrosine kinase domain, are able to establish a vascular tree, and survive embryogenesis, it appears that VEGFR1 signaling is not required per se for endothelial cell development. However, the VEGFR1 extracellular domain serves to bind VEGF and to negatively regulate VEGFR2 activity. The mechanism whereby the three ligands for VEGFR1, VEGFA, VEGFB, and PlGF, exert their different biological effects is unclear but could involve differential binding to additional receptors or co-receptors, such as the neuropilins (15).

VEGFC and VEGFD bind to VEGFR3. VEGFC gene inactivation results in disruption of lymphatic vessel development and prenatal death due to tissue fluid accumulation (21). VEGFD gene targeting, on the other hand, is compatible with normal mouse development (22). Knock-out of VEGFR3 leads to a distinct phenotype consisting of cardiovascular failure and embryonic death at E9.5 (23). VEGFR3 is expressed in tip cells of sprouting vessels, in agreement with an important role for VEGFR3 in angiogenesis (11). VEGFC as well as VEGFR3 have been implicated in induction of tumor angiogenesis and lymphangiogenesis (24). Integrins can induce Src-dependent phosphorylation of VEGFR3 and downstream signaling independently of VEGFC/VEGFD and activation of the VEGFR3 kinase (25).

#### VEGFR signaling

VEGFR tyrosine phosphorylation patterns have been described *in vitro* (26-28), and the *in-vivo* validation for certain of these sites has been initiated (29). VEGFRs share many signaling pathways with most if not all receptor tyrosine kinases, such as the phospholipase Cγ (PLCγ) pathway regulating proliferation of endothelial cells through the extracellular regulated kinase (ERK) pathway and the phosphoinositide 3' kinase (PI3K)/AKT pathway. For VEGFR2, which is the most studied VEGFR this far, the following features stand out: 1) Ras activation may not be induced in response to VEGF; instead induction of proliferation is dependent on PLCγ/ERK. In agreement with this notion, a tyrosine-to-phenylalanine knock-in at Y1173, the VEGFR2 phosphorylation site that binds PLCγ, leads to embryonic death and endothelial cell deficiency (29). 2) VEGFR2 activates endothelial nitric oxide synthase (eNOS) which is important in regulation of vascular permeability (30). Another potential pathway in regulation of vascular permeability involves the VEGFR2 phosphorylation site Y949, which binds the adaptor molecule T cell-specific adaptor, which in turn promotes activation of Src, allowing regulation of endothelial junctions (28). For a comprehensive review on VEGFR signaling, see Koch et al. (13).

#### Diseases and therapies

VEGFA production is enhanced in hypoxia and is therefore found in growing tissues such as cancer. Although VEGF is not a biomarker in cancer, it is expressed by most, if not all, forms of human tumor disease. A recent focus has been on inflammatory cells, which infiltrate the tumor and may constitute most of the tumor mass. Inflammatory cells are important in delivery of angiogenic growth factors, such as VEGF, to the tumor (31). A critical question is whether VEGFRs are expressed not only on tumor endothelial cells but also on the tumor cells, where VEGFR regulation and signaling may be distinct from that in endothelial cells. Therapy strategies developed this far are, however, based on neutralizing antibodies or kinase inhibitors and independent of the expression pattern of VEGFs and VEGFRs (32). Clinically more pressing questions are instead those of side-effects and refractoriness/resistance to treatment, as discussed below.

In 1993, Kim et al. showed that a neutralizing antibody against mouse VEGF (A.4.6.1) inhibited tumor growth and angiogenesis in mouse models (33). The positive results obtained with A.4.6.1 led to the development of a humanized version of this antibody, bevacizumab (Avastin), providing one of the most successful marketed compounds within the anti-angiogenic therapy field.

DC-101, a monoclonal antibody that targets murine VEGFR2, efficiently blocks tumor growth in a variety of tumor xenograft models as well as hepatic metastasis derived from colon cancer (34,35). Fully humanized anti-VEGFR2 antibodies were developed for subsequent clinical use, such as IMC-1121B (ramucirumab). Studies performed with this antibody have demonstrated its efficient anti-tumor effects in murine xenograft models (36,37).

Several tyrosine kinase inhibitors (TKIs) that inhibit VEGFR2 have been tested in preclinical studies. Sunitinib (Sutent; Pfizer), sorafenib (Nexavar; Bayer), and pazopanib (Votrient; GlaxoSmithKline) are the most advanced drugs within this group.

### Fibroblast growth factors (FGFs)

#### Ligands

The FGF family encompasses 22 proteins (FGF1–23) identified to date (38). FGF2 is a potent mitogen for endothelial cells *in vitro*; it was the first angiogenic growth factor to be identified (39). FGFs have several distinguishing features. Thus, although most FGFs are secreted, the prototypic FGF1 (acidic FGF) and FGF2 (basic FGF) lack a signal sequence typical for secreted proteins. The mechanism for secretion of FGF1 and FGF2 has remained controversial, although several mechanisms have been suggested, such as direct translocation across the plasma membrane or heterodimerization with carrier proteins (40). Alternatively, FGF1 and FGF2 become released upon cell death. Furthermore, certain FGF variants are taken up and brought to the nucleus, where they may play a role in regulation of proliferation (41).

#### Receptors

There are four FGF receptor tyrosine kinases (FGFR1–4) with a very similar molecular organization of three extracellular Ig-like loops, a transmembrane domain, and a split tyrosine kinase domain (Figure 2). The extracellular FGFR domains undergo extensive alternative splicing, which regulates ligand binding. There is a certain degree of tissue-specific expression of the different splice variants, which is matched by the tissue-specific expression of the corresponding ligand. However, FGF1 will bind all FGFR isoforms (see (42) for a comprehensive review).

FGFs bind with very high affinity to HSPGs, which are known to act as co-receptors for the FGFRs (43). FGFs have also been reported to bind to neuropilins, but the details of the binding motifs and the biological consequence remain to be described.

#### Lessons from genetic models

A challenge in deciphering the biology of FGFRs has been their low expression levels, typically around 1,000 molecules/cell. Whether any of the FGFRs indeed are expressed in endothelial cells *in vivo*, possibly in specific organs or in particular processes, has been difficult to determine. Gene targeting efforts have established that FGFs/FGFRs have essential roles during development and in a wide spectrum of physiological processes such as skeletal development (44). In contrast, accumulating data have not supported the notion that FGFs/FGFRs act directly on EC *in vivo*. That FGFRs are expressed on endothelial cells *in vitro* and that FGF2 is a strong mitogen for endothelial cells in culture are well established. FGFs/FGFRs may still be very important for vessel biology *in vivo*, e.g. by regulating expression of cytokines that in turn may affect vessel function. For example, FGFR1-deficient embryonic stem cells show an exaggerated tendency to form EC, due to a FGFR1-dependent change in expression of cytokines such as interleukin-4 and pleiotrophin (45). Furthermore, FGFs act directly on EC in pathologies such as cancer where FGFRs may become up-regulated (46) (see below).

#### FGFR signaling

FGFRs are first and foremost associated with cell proliferation. The adaptor protein FRS2 (FGFR substrate 2) acts as a hub linking several signaling pathways to the activated FGFRs (42). FRS2 binds to the juxtamembrane region of FGFR, and upon activation of the receptor it becomes phosphorylated on several tyrosine residues, creating docking sites for additional adaptor proteins, including the Ras/ERK pathway and the PI3K/AKT pathway.

#### Diseases and therapies

Elevated levels in serum of FGFs and FGFRs have been described in many forms of human cancer, including brain cancer, head and neck cancer, gastric cancer, ovarian cancer, and bone cancer. The spectrum of target cells for the ensuing activities is likely to be broad, including endothelial cells.

Several TKIs that inhibit FGFRs are tested in early-phase clinical trials; none of these is specific for FGFRs, and often also VEGFRs and PDGFRs are targeted. Novel more specific FGFR inhibitors are being developed at present, such as AZD4547 and BGJ398 (see http://ClinicalTrials.gov).

### Platelet-derived growth factors (PDGFs)

#### Ligands

PDGFs, which are structurally related to VEGFs, occur as covalently linked homo- or heterodimers. There are five dimeric PDGF isoforms, assembled from polypeptide chains denoted A, B, C, and D (i.e. PDGF-AA, PDGF-BB, PDGF-AB, PDGF-CC and PDGF-DD) (47). The A and B forms are secreted as active forms, whereas the C and D forms are produced as inactive forms containing CUB-domains which must be removed to allow receptor binding (47). The different PDGFs are produced by a variety of epithelial cells.

#### Receptors

PDGFs interact with two structurally related receptor tyrosine kinases, denoted PDGFRa and PDGFRb, which may homo- or heterodimerize depending on the ligand. The receptors are structurally highly related, with five Ig-like extracellular loops, and a split tyrosine kinase domain (Figure 2). Both receptor types are expressed on mesenchymal cells (48). Interestingly, PDGFRb expression is regulated by inflammation. Thus, fibroblasts in healthy tissues do not express PDGFRb, whereas fibroblasts in tissues involved in inflammatory processes such as rheumatoid arthritis express the receptor, as do cultured fibroblasts (49).

PDGFA and B forms bind HSPGs/heparin, but with relatively low affinity (50). Integrins are critical modulators of PDGFR signaling, and adhesion of cells to specific extracellular matrix may activate PDGFRs in the absence of a ligand (51).

#### Lessons from genetic models

Overall, PDGF has important roles during development in particular of mesenchymal cells. In the adult, PDGF contributes to wound healing and regulates interstitial pressure (48). Different PDGFs and receptors have been studied by gene inactivation in different combinations. PDGFA deficiency gives rise to lung emphysema due to a defect in alveolar smooth muscle cells (52). Mice with PDGFC-deficiency die at birth due to respiratory failure and cleft palate, which impairs suckling (53). Combined deletion of PDGFA and PDGFC genes has effects resembling those seen in mice lacking PDGFRa (54). Inactivation of PDGFD does not result in a phenotype in unchallenged mice (personal communication, Professor Ulf Eriksson, Karolinska Institute, Sweden). None of these genes directly affect endothelial cell biology. Gene inactivation of PDGFB and PDGFRb (55–57), on the other hand, gives rise to very similar phenotypes, with loss of mesangial cells in the kidney and close to complete loss of pericyte formation. Loss of pericyte formation results in a weakened vessel wall and hemorrhaging, resulting in perinatal death.

#### PDGFR signaling

PDGFRb was one of the first receptor tyrosine kinases to be studied in detail, by mapping of phosphorylation sites and identification of Src Homology 2 (SH2) domain proteins and other signal transducers binding specifically to the different sites. There is therefore detailed information on the pathways induced, which include all major known signaling pathways such as the PLCγ/PKC, PI3K/AKT, and Ras/MAPK pathways. A similar pattern exists for PDGFRa (48).

Interestingly, PDGFR signaling may be modulated by VEGF. It was recently suggested that the effect of PDGF on pericytes is negatively regulated by VEGF through formation of VEGFR2/PDGFRb complexes (58).

#### Diseases and therapies

PDGF over-activity has been linked to tumor progression as well as to atherosclerosis and fibrotic conditions, and it is clear that PDGF signaling may contribute to multiple tumor-promoting processes. Moreover, gene rearrangements, mutation, and amplification of PDGF or PDGFR family members have been linked to relatively rare forms of cancer such as gastrointestinal stromal tumors (48).

Tumor vessels often show incomplete pericyte coverage, which at least in part contributes to the poor functionality of the tumor vasculature, with poor perfusion, leakiness, and turnover. Moreover, PDGFRb expressed on stromal fibroblasts regulates interstitial fluid pressure, which is elevated in tumors, interfering with efficient uptake of drugs (59). Expression of PDGFRb on tumor pericytes has allowed targeting of pericytes in cancer therapy, and preclinical analyses have indicated that combined anti-VEGF and anti-PDGF therapy may be beneficial (60).

## The role of VEGF, FGF, and PDGF in refractoriness to anti-angiogenic treatment

Despite the successes of benchmark therapies like Avastin/bevacizumab (anti-VEGF antibodies), not all patients benefit from treatment. In some patients, tumors shrink only to grow again, and the clinical benefit is usually measured in months, not years. A number of phase III clinical trials have shown no benefit (61). Moreover, in preclinical models, suppression of VEGF has been associated with more aggressive disease and increased metastatic spread (62,63). Several mechanisms have been brought forward to explain the refractoriness to anti-angiogenic treatment (64), as outlined below. A consistent theme is the plasticity of the tumor.

### Advanced disease

With progressive disease, the sensitivity to anti-angiogenic therapy diminishes (65). There may be no response, or an initial response followed by relapsed disease. Advanced cancer may have reached a state where a wide range of growth stimuli overlap in their promoting effects on different cellular constituents. Moreover, the tumor cells have undergone a number of mutations and chromosomal rearrangements, further aggravating the disease.

### Alternative mechanisms in blood vessel formation

Blood vessels may form through several mechanisms that may be differently regulated (see above). Whereas a wealth of data implicate sprouting angiogenesis in tumor vascularization, intussusception, co-option, and vasculogenic mimicry may also be involved. Formation of vascular channels lined by cancer stem cell-derived endothelial cells has been shown, e.g. in glioblastoma (66).

### Production of angiogenic growth factors and endothelial cell plasticity

With time and in a situation where VEGF or VEGFRs are inhibited, endothelial cells may acquire the capacity to respond to other, compensatory growth factors such as FGF2, stromal derived factor 1 (SDF1), ephrins, angiopoietins, and PDGFB and PDGFC (67–69). These growth factors may be produced by tumor cells, by bone-marrow-derived cells (BMDCs), tumor-associated macrophages (TAMs), or tumor-associated fibroblasts (70). For example, treatment of tumor-bearing mice with an anti-VEGFR2 antibody resulted in an initial decrease in vascularity followed by vascular rebound and in an increase in expression of FGFs (67). Increased levels of circulating FGF2 have also been noted in human patients treated with AZD2171, a pan-VEGFR2 tyrosine kinase inhibitor (71). PlGF was also reported to be increased in the circulation following treatment with sunitinib (72).

### Epigenetic and genetic mechanisms in resistance

Genetic and/or epigenetic patterns in tumor endothelial cells are unstable and change after anti-angiogenic treatment (73,74). The original concept that endothelial cells are ‘normal’ and derived from healthy vessels that are stimulated by production of VEGF in the tumor is probably only partially correct. For example, endothelial cells in healthy tissues do not express receptors for epidermal growth factor (EGF); however, in cancer forms that express EGF, such as prostate cancer, EGFR is expressed on tumor endothelial cells (75).

## Is anti-angiogenic therapy a viable long-term prospect in cancer treatment?

Although the introduction of anti-VEGF therapy in cancer was coined a breakthrough, and many patients have had clear benefits of the treatment, there is more to do to optimize both the treatment itself (dose, time-frame, combination with chemotherapy). It has also become clear that while large cohorts of patients with advanced disease may not benefit from the treatment, there are groups of individuals for whom this treatment is very important. Two major lines of efforts are important in the future development of anti-angiogenic therapy.

### Preclinical studies versus human disease

Tumor models in mice lack most of the characteristics typically found in human disease. Preclinical studies are predominantly performed with tumors that grow in the avascular subcutaneous compartment. Here, tumors have to induce angiogenesis in order to grow, thus being more susceptible to anti-angiogenic therapy. In human cancer, however, tumors often grow in highly vascularized tissues, where growth not only depends on angiogenesis but also on vessel co-optation, thus increasing resistance to anti-angiogenic compounds. Moreover, for most tumor models in mice, the disease is acute, whereas in humans, it arises slowly over the course of many years or decades. Finally, in mice, the study is interrupted due to ethical concerns based on the properties of the primary tumor irrespective of metastatic spread. In humans, it is the metastatic spread that presents the major threat. Characteristics that clearly distinguish metastases from primary tumors have been very difficult to define, due to the molecular heterogeneity of the metastatic lesions. It is also unclear if hematogenic and lymphatic spread of tumors should be differently treated or targeted. The standard for tumor models has to be clearly defined in order to be relevant for human disease.

### Early treatment and identification of biomarkers

VEGF inhibition in preclinical models is accompanied by a window of vessel normalization, which may facilitate for example delivery of chemotherapy. To exploit this aspect in the treatment of human cancer, current imaging and non-invasive markers of maturation must be further developed. Moreover, combinatorial therapy with chemotherapeutic agents needs further refinement, since chronic angiogenesis inhibition reduces tumor uptake of co-administered chemotherapy. Overall, based on the collected experience, improved strategies for clinical trials need to be developed. Efficient anti-angiogenic therapy for human cancer requires biomarkers to select responders within the patient cohorts. Clearly, efficient anti-angiogenic responsiveness depends on the stage of tumor development and on the tumor microenvironment. Individualized therapy will be a key to future successful development of cancer therapy.
